# A peripatetic pediatrician's journey into pediatric rheumatology

**DOI:** 10.1186/1546-0096-5-11

**Published:** 2007-05-23

**Authors:** Earl J Brewer

## Abstract

Earl Brewer discusses his journey into pediatric rheumatology from 1958 to retirement in 1990 in three parts.

## Part I: the early years

### Introduction

#### Who is the father of pediatric rheumatology?

**Figure 1:**
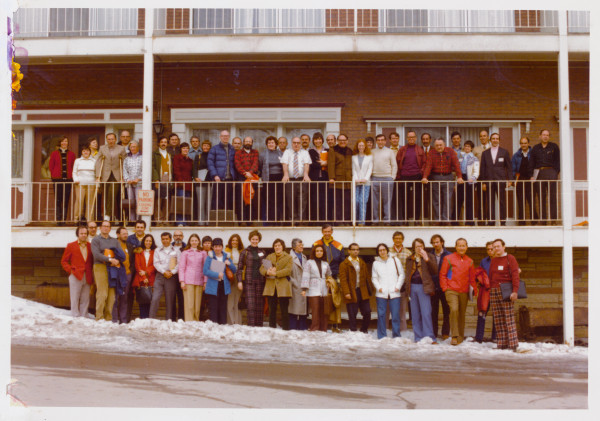
Fifty-nine participants pictured at Treasure Mountain Inn, Park City Utah, 1976.

"I think I saw it arrive, although I cannot specify its birthday or place and I am damned if I can read the father's signature on the birth certificate. We are very fortunately a small enough group to know each other personally and to cooperate and enjoy each other's company." Eric Bywaters, Park City I Meeting. 1976, [figure 1].

"Thirteen physicians including two Nobel Laureates in 1932 held the first meeting of the American Committee for the Control of Rheumatism. In five years it became the American Rheumatism Association (ARA). I first attended the seventh meeting of the ARA in June 1940. Only five papers were presented. Three (60%) of the papers were devoted to children with rheumatic disease. One of the papers was by Dr. William Green, an orthopedist at the Children's Hospital in Boston. His subject, "Mono-articular and Pauciarticular Arthritis in Children." J. Sydney Stillman, Park City II Meeting. 1986

"In the beginning we were a handful of naïve but eager and explorative, young physicians of disparate training, background, and temperament. We joined together with a vision of doing whatever was necessary to find better ways to study the rheumatic diseases of childhood and adolescence, and to treat our patients more effectively in a comprehensive continuum of care." Earl Brewer and Joseph Levinson, Foreword, Textbook of Pediatric Rheumatology. 2005

As I enter my seventy-ninth year, my attention turns to committing to paper, memories and thoughts about my professional journey into what we now call pediatric rheumatology. Since my retirement seventeen years ago, several colleagues and friends have encouraged me to describe the road on which we all traveled to provide better care and research for children with arthritis.

My approach will be to share personal memories and thoughts about how I came to the table and what happened along the way. There will be no attempt at a global history of our field and no attempt to impartially tell the story of others who labored so diligently to help the children. Landmark concepts, occasions, or events in pediatric rheumatology will be identified, and the development of each concept or event carried forward.

I shall share my thoughts in the first person as a writing technique, which limits my scope to what I personally know or have been told. I carefully kept records of the journey from 1958 to 1992. For the interested reader, the papers are located in the, John P. McGovern Historical Collections and Research Center, Houston Academy of Medicine, Texas Medical Center Library, 1133 John Freeman Blvd., Houston, Texas 77030-2809,. For further information call 01-713-799-7145. The collection is housed in 48 boxes that occupy over 48 cubic feet. They are available by appointment through Ms. Elizabeth White, Director of the McGovern Center. The finding aid is also digitized on the Internet site of the library under manuscripts.

My partner along the way, my wife, Ria, has always been integral to all of the steps on the journey. I dedicated four books to Ria with the lines, "To Ria, the best of the best. With such apparent ease, she makes everything possible."

#### Overview

There were three ten-year plans for the development of pediatric rheumatology from 1958 to 1990.

*The 1960's: *Our efforts started when the old March Of Dimes became interested in arthritis. They applied the lessons learned from polio. The first special treatment centers for children with arthritis started in 1960. Now we needed a common base to talk to each other. The American Rheumatism Association appointed the JRA Subcommittee of the Classification Committee in 1964. This event persuaded several of us in England and the USA to fill out lengthy questionnaires concerning the clinical course of children we all thought had JRA. The resulting data allowed preparation of what we called "JRA Criteria" published in 1972. The database was subsequently expanded and checked for usefulness.

*The 1970's*: With the criteria as a stepping-stone, it became clear that we needed drugs to treat children with JRA. We developed a methodology to study medicines for children with JRA. This led to the formation of the Pediatric Rheumatology Collaborative Study Group in 1973. The next step was to develop a council to have a voice in the affairs of the ARA. The council's first project was a meeting of physicians interested in pediatric rheumatology. The Arthritis Foundation, ARA, and the Shriners of North America sponsored the meeting in Park City, Utah in 1976. The first supplement devoted to pediatric rheumatology was the best selling supplement in the history of the Arthritis and Rheumatology journal. [1]

An unexpected benefit was our inclusion in international rheumatology by way of the USA-USSR scientific cooperation program supervised by the National Institutes of Health in 1975. Ties with colleagues in Europe led to an international pediatric rheumatology meeting in Oslo in 1977.

The 1980's: The American Academy of Pediatrics was persuaded to form a pediatric rheumatology section of the AAP in 1980. Attention was needed to develop centers to give coordinated care for children with arthritis. The Maternal and Child Health Bureau of the US government selected pediatric rheumatology for development. We joined forces with them to form many regional pediatric rheumatology centers under a special program (SPRANS-Special Programs of Regional and National Significance) and develop a host of innovations. The Bureau sponsored a pivotal meeting in Houston in 1984, *New Horizons in Pediatric Rheumatology*.

1984 was a busy year in that we also had the first American Juvenile Arthritis Organization meeting in Keystone, Colorado, under the auspices of the Arthritis Foundation.

The last of the eighties was perhaps the most satisfying. We petitioned for a board of Pediatric Rheumatology in 1980, and in 1990 the American Board of Pediatrics and the American Board of Medical Specialties formally approved formation of the Board.

#### The early years

**Figure 2:**
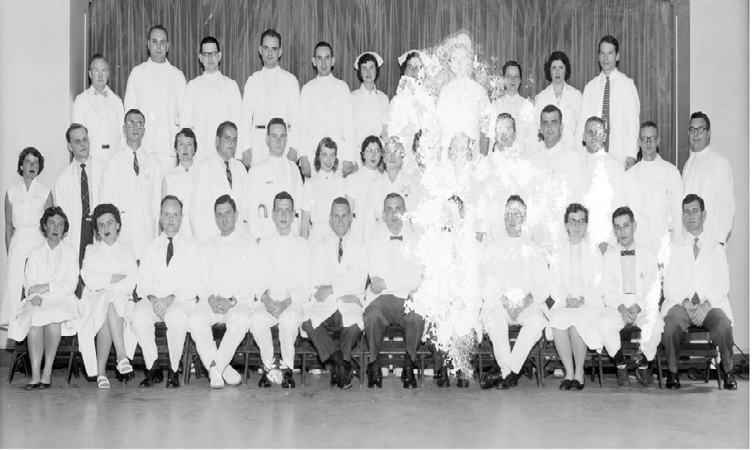
1957 House staff – children's hospital – Boston. Brewer is fifth from left, front row. Dr. Janeway is sixth, Dr. Peter Auld is seventh, Dr. Fred Rosen is last on right, second row, Dr. Joel Alpert is fifth from left, top row.

My first exposure to the problems of children with arthritis occurred when I was a Senior Resident in Medicine at the Boston Children's Hospital in 1956 and1957. [figure 2] Dr. Charles Janeway was Physician-in-chief and Chair. The six senior residents met with him at eight each Monday morning in his office in the old hospital building on the second floor. We discussed what had happened in the hospital over the weekend. My first experience at the Boston Children's Hospital was like Crocodile Dundee coming to the United States. My previous training was at Baylor University College of Medicine and the pediatric program in Houston. The journey to Boston was my first trip away from Texas except in the army in California. I was not just wet-behind-the-ears, I was very wet everywhere. I was six feet three inches and weighed about 130 pounds. I was not just skinny – I looked anorexic. I did have a nice smile. My professional journey from Houston to Boston is interesting in itself and will be discussed later.

The hospital program had six senior residents and twenty-four junior residents. Three of the senior residents were selected from the junior resident group each year, and three senior residents were from the outside. Dr. Janeway wanted to prevent inbreeding of the program and used this method of germinal seeding.

I was one of the three outsiders. After we became friends later in the year, Bob Berg, a fellow senior resident and homegrown Harvard man, asked how it felt to have overcome an inferior educational background and made it to the big time. Texas had not qualified as big time at that juncture east of the Charles River.

One Monday, Peter Auld, the chief resident, and Dr. Janeway lamented how the children with JRA were so poorly treated. Dr. Janeway was concerned about the children receiving long-term hospitalization at the rheumatic fever hospital, the House of Good Samaritan. Rheumatic Fever was rapidly becoming history because of penicillin, and the children with JRA were housed at the "Good Sam." The hospital was part of the Children's complex, and the senior residents covered the needs of the Good Sam on an as-needed basis.

I was assigned the task to review all of the children who had been admitted to the hospital, mainly in the affiliated House of Good Samaritan, over the years. I spent many hours reviewing voluminous charts. The idea was to write a review paper and show how many problems existed with these children and how we needed better ways to treat them. The records were not uniform, and gathering decent data were not possible. In general, many of the children were put to bed and many just shriveled in size and stature. A genesis of the interest in the care of these children was the perceived rivalry with the Massachusetts General Hospital adult rheumatology program under Dr. Marion Ropes. The pediatricians felt that the adult physicians' approach was not what we would like. I reluctantly told Dr. Janeway that the records could not be used properly. The time I spent reviewing these children's charts, however, left me with a nagging worry about the care of these children. I also learned a valuable lesson from the records: **One must always make sure that an adequate and planned record is made of clinical material. This lesson was key to whatever success occurred later in my career**.

#### Texas Children's Hospital, Baylor College of Medicine, and Kelsey Seybold Clinic

In 1958 I returned to Houston as Chief Resident at Texas Children's Hospital by prior arrangement as part of my Jesse H. and Mary Gibbs Jones fellowship to Boston agreement. The hospital was only four years old and in its infancy as an institution. Dr. Blattner was chair and physician-in-chief. He was my mentor and friend. His broad vision made the hospital and pediatric department a huge success during his tenure. He promoted the idea that parents should stay in the hospital room and participate in the care of their children. The parents were only allowed to visit at certain times of the day at other Children's Hospitals.

He could not afford to hire expensive, established specialists for his department, so he sent promising young people to other centers in the country and brought them back to Houston. I spent the year as chief resident innovating such things as the development of our resident teaching conferences and a system of discharge summaries. I also learned many lessons in my attempts to integrate effective interaction between the private physicians and the resident staff. The hospital became incredibly busy, and our 100 beds were woefully inadequate. One major program consumed much of my personal interest. Dr. Dan McNamara and Dr. Denton Cooley were developing their now world-famous cardiology program. Dr. Blattner allowed me to spend two months with Dan as a fellow in cardiology. At the end of my chief residency, I contemplated joining the Cardiology section, but economic circumstances dictated private practice in the nearby town of Wharton with Dr. Bolton Outlar, also a friend and mentor.

It was at this point that Dr. Blattner discussed my future and how I could best participate in academic medicine, research, and practice. The evolution of the Texas Children's Hospital/Baylor College of Medicine arthritis program began in early 1958 when Dr. Blattner and I began a series of talks concerning treatment of children with rheumatoid arthritis. My nagging concern about these children persisted since my time in Boston. Rheumatic fever hospitals closed as penicillin treatment and time reduced the incidence. We were concerned that the children with juvenile rheumatoid arthritis (JRA) were not being treated properly. We learned from Dr. William Spencer's work with polio that active exercise was always better than excessive rest. Dr. Blattner asked me to look into the situation and begin an arthritis program at TCH. We promoted the concept of keeping the children at home and in school with hospitalization almost always a last resort. We also promoted the idea of keeping children as active as possible even when pain was present.

Dr. William Clark in 1958 took over the March of Dimes when they were searching for a new disease to solve after conquering polio. Dr. Blattner and I spoke with Bill Clark and told him of our program of strengthening muscles and continued active exercise and activity because of data showing that enforced rest results in atrophy and weakness, not improvement. He understood and invited me to his Vanderbilt meeting in Nashville in 1959 to discuss his plans for the March Of Dimes (more about that meeting in a separate section).

The clinic took place one morning each week in the Junior League Clinic of the Texas Children's Hospital in 1958 with myself as the physician, a nurse and volunteers in attendance. Medical students, pediatric residents, and nursing students were assigned to the clinic for that particular morning of the clinic. Within a short time the March Of Dimes Special treatment Center was funded. Our own physical therapy room was approved and was essential to our success.

The program was a strictly a shoestring operation. I practiced an hour away from the hospital in a small town and came to TCH one day a week for the program. The addition of our own physical therapy room illustrates the primitive structure of our efforts. Dr. Blattner and I shared offices on the main floor of the hospital just off the modest lobby. The personnel director had a private bathroom at the end of the hall, which infuriated Dr. Blattner and Leopold Meyer, Chairman of the Board of the hospital. Dr. Blattner and I sneaked into the room one day to measure the space to see if we could put our just approved physical therapy unit there. Lo and behold, Mrs. Smithson, the personnel director, came into the bathroom, and we hid in a stall. She never found us, and we liberated the space for our new effort.

The early structure of our approach was pragmatic, and we dealt with the resources at hand. Health insurance in Texas would not pay for prolonged hospitalization of these children. A major objective of the clinic was to provide long term ambulatory care to not only the child but also the family through the duration of illness. Another major objective of treatment was reduction of pain and discomfort, prevention of deformities, and maintenance of general health and well-being.

A landmark event occurred when I was allowed to hire Mrs. Elizabeth Barkley In 1960 as our first physical therapist through a grant from the March Of Dimes (more later about the MOD). Elizabeth previously was director of the physical therapy school at the Hermann Hospital in the Texas Medical Center. She was also a member of the Texas Board of Physical Therapy. My good fortune was that she shared our missionary zeal to do our best to improve care for the children with arthritis.

We developed what we termed a Home Treatment Program (HTP). A consulting orthopedic surgeon was added about this time. The children were evaluated in the clinic, and a treatment program of daily, active, planned exercise and heat at home was planned. Two or three visits to our hospital physical therapy department each week were not sensible or economically possible for a child with a chronic condition. The program consisted of parental education with emphasis on the daily exercise routine of the patient under the guidance of the parent. The child and parents were instructed in the daily heat and exercise program and were checked at weekly intervals by the physical therapist. When the program was fully understood and established, the child and parent were checked only at regular clinic visits. Unfortunately our only medications were aspirin and steroids, and steroid use was in an early stage and was poorly understood.

The modalities used were warm tub baths twice each day followed by a formal exercise program. These exercises were adapted to fit the needs of the individual child and designed to prevent atrophy and maintain range of motion. Active exercise was encouraged as opposed to passive exercise. In situations of severely involved joints, active, assistive exercise was employed to maintain normal range of motion. Play activities that could be carried on for a long period during the day and produce the desired strengthening and range of motion were outlined for the child. Night splints were used sparingly to increase range-of-motion.

An important aspect of the program was emphasis on encouraging the child to carry on ordinary activities at home and school. The parents and siblings were grounded in treating the child as any other member of the family including doing chores.

Our companion instructional booklet, The Home Treatment Program, for parents and children with arthritis was published in the early 1960s and received national and international recognition [2]. All of us were pleasantly surprised at the improvement shown by the children who followed our program. It was clear to me that active exercise is almost always better than the then recommended prolonged bed rest regimens.

#### Early interaction with adult rheumatology world

From the beginning, a perceived tension existed between the adult rheumatologists trained in internal medicine and pediatricians beginning to care for these children. There were fundamental differences in training and approach to the care of children and in particular, children with arthritis. Each group felt self-righteous and absolutely sure that its view was correct. It should be mentioned that the American Academy of Pediatrics (AAP) was not organized until the early 1930s. The AAP was created largely because the American Medical Association (AMA) refused to support the formation of The Children's Bureau, and physicians fighting for immunizations for children split from the AMA. A separate department of pediatrics was not formed at Baylor until Dr. Blattner was hired in 1947. The scene was set with a background of distrust on the part of the pediatricians protecting their newly created independence and a perceived patronizing attitude of the adult rheumatologists toward the pediatricians.

I first attended a meeting of the then American Rheumatism Association in the late 1950s. I attended with my new friend, Dr. James Kemper. He had recently arrived in Houston from the Mayo Clinic to be in charge of rheumatology in the Department of Medicine at Baylor and rheumatologist at the then small Kelsey Seybold Clinic. Fortunately for me and my career in pediatric rheumatology, we both liked each other from the beginning and began a professional life and friendship together that has lasted until now.

The whole experience was incredible. I enjoyed learning about the huge world of arthritis, and I was impressed with the caliber of the adult rheumatologists. I also came away with the certain knowledge that the adult physicians had a frame of reference with regard to care that was fundamentally different from pediatricians. It was more than the usual cliché that children are not little adults, although that was certainly one facet of the differences. Another cliché was that if internists wanted to take care of children, they would have become pediatricians. Clearly, there were internists who loved caring for children also.

I met Dr. William Clark, who trained at the Massachusetts General Hospital with so many legends of rheumatology including Dr. Ronald Lamont-Havers, then with the Arthritis Foundation in New York, Dr. Sydney Stillman, Director of the Robert Brigham Hospital at Harvard, Dr. John Ward of Salt Lake City, and Dr. Morris Ziff, another legend of rheumatology. Sydney Stillman ran a children's clinic at the Robert Brigham Hospital in Boston, but he did not have a close relationship with the Boston Children's Hospital. He was one of the great men I have known and became a mentor for me. I wish I had known him when I was a resident in Boston.

However the conclusions that I drew from my early, limited experiences were clear to me. The path to better care and research for children with arthritis had be a joint journey with the adult rheumatology world but must be led by pediatricians. I followed this concept until my retirement in 1990. The concept led to some memorable battles along the way.

In the late 1950s several other pediatricians and internists were devoting time to caring for children with arthritis in several parts of the country. We all became devoted colleagues in our quest for better care and research for the children with arthritis. Others will hopefully tell the stories of the early beginnings of their clinics and their journeys into pediatric rheumatology.

#### The pioneers

The experiences related in my journey were shared completely with a large number of dedicated legends of our field along the way. A few impressions are given here about the pioneers who began in the 1950s:

##### Dr. James Cassidy, Ann Arbor–University of Michigan, Omaha–Creighton Medical School, Columbia–University of Missouri [figure 3]

**Figure 3:**
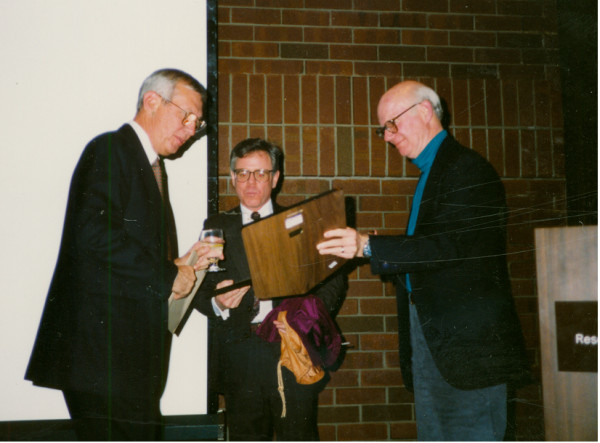
Dr. James Cassidy [R], Dr. Earl Brewer [L], Dr. Edward Giannini [C].

I can't remember when I first met Jim, but we were friends from the beginning. He was a type A, rapid-thinking but careful physician. Jim was our resident genius and writer. His gentle smile and a humorous gleam in his eye added to his disciplined approach to any task. Jim was prematurely bald and was famous for his caps. He was trained in internal medicine at the University of Michigan and later passed boards in both medicine and pediatrics. He became Chair and Professor of Pediatrics at Creighton University and moved to the University of Missouri later and limited his rheumatology patients to children. He was well known among his peers for eating dinner at precisely six in the evening for reasons unclear to any of us. We have remained close friends and colleagues over the years.

Jim, among many accomplishments, is the co-author with Ross Petty of the standard Textbook of Pediatric Rheumatology published many years later. He and his wife, Nan, were high school sweethearts in Oil City, Pennsylvania. He was a consensus builder in our group. His interest in children with arthritis started at Michigan during his training. He felt no one was looking after the children. He encountered resistance from his adult rheumatology staff from the beginning and had to make sacrifices to continue. He felt the same need the rest of us felt and became a staunch and extremely important pioneer in our efforts.

##### Dr. Joseph Levinson, Cincinnati Children's Medical Center, University of Cincinnati [figure 4]

**Figure 4:**
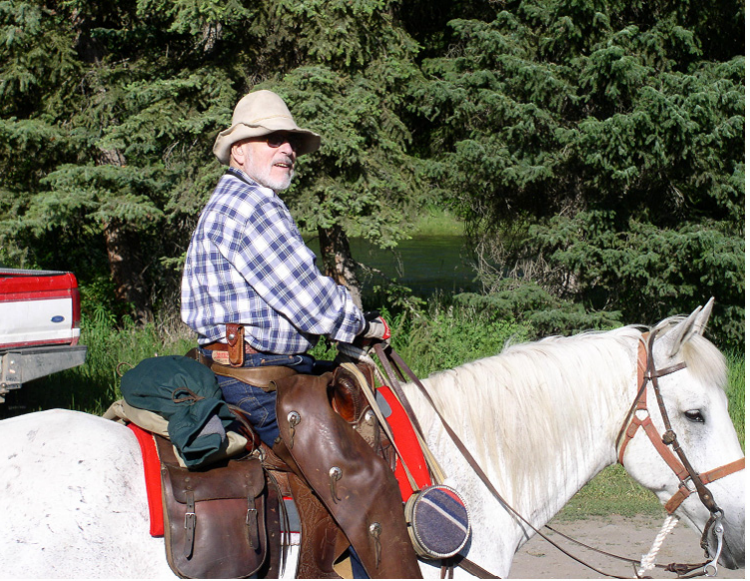
Dr. Joseph Levinson.

Joe Levinson is another giant legend of our field. We were so fortunate for his presence in our small group. He was a reflective, Talmudic person. (Talmudic scholarship is famous for the tortuous and painstaking manner in which truth is pursued and established–if it can be established at all.) Joe would agonize over a single word included in our various publications. Jim Cassidy and I at times threatened him with extinction if he didn't finish editing a given paper and give approval. Joe trained in adult rheumatology at the MGH in Boston. He entered pediatric rheumatology almost by chance. He filled in at a pediatric clinic in Cincinnati, which has become a premier program of pediatric rheumatology. His vision for his center-to-be was incredible. His persona filled the room when he entered. He was a jolly-everyone's-Dr. Joe. His soft-spoken words flowed with ease. His physical stature was spare. He was and is truly a gentle man.

##### Dr. Chester Fink, Dallas, Southwestern Medical School, Scottish Rite Hospital [figure 5]

**Figure 5:**
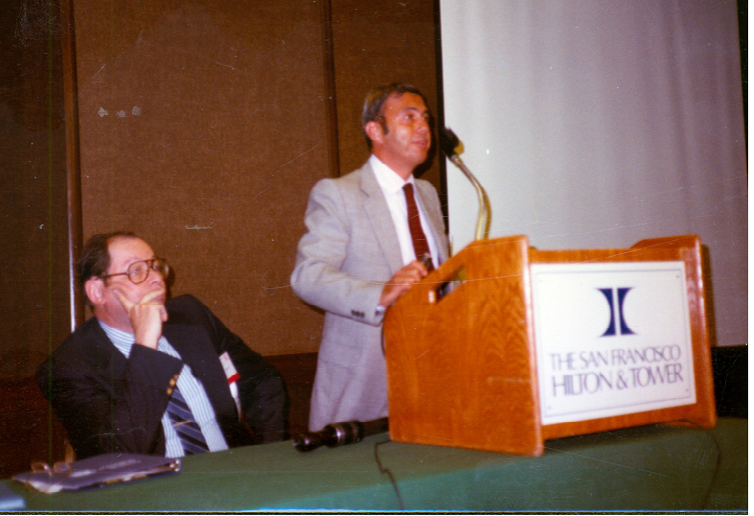
Dr. Jerry Jacobs [R] and Dr. Chester Fink [L].

Chester Fink was another legend and a wonderful pediatrician from North Carolina who trained at Case Western Reserve and was a friend of Bill Clark. Chet settled at Southwestern Medical School in Dallas and started a clinic at the Scottish Rite Hospital for Children and worked with Morris Ziff, head of the adult rheumatology program and another legend of rheumatology. Chet loved to eat and was pleasantly plump. His sense of humor was a pleasure for all of us. His quiet and reflective approach to the problems at hand always made sense. His cheeks were red, topped by horn-rimmed glasses. His ready smile and laugh lightened many a tense situation, as each of us pressed our personal agendas during our deliberations. He became a great friend. Chet met his wife, Dotty, when they were both stationed in Japan in the army. They retained their love of Japan and Chet was our main contact person for Asia in our collegial press to develop better care for children with arthritis.

##### Dr. Virgil Hanson, Los Angeles, Children's Hospital of Los Angeles, University of Southern California [figure 6]

**Figure 6:**
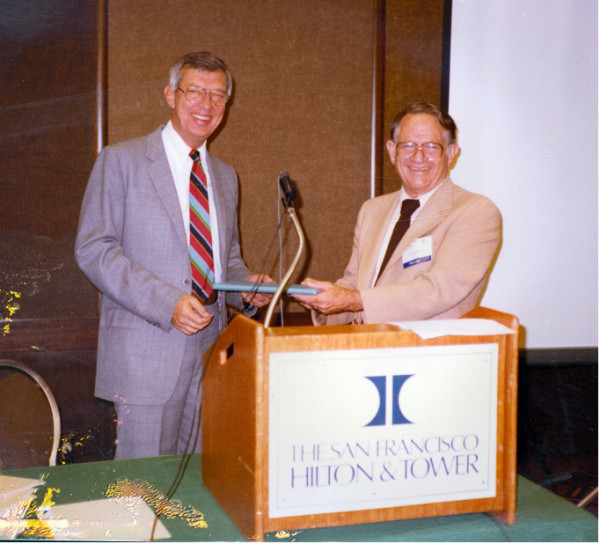
Dr. Virgil Hanson [R] and Dr. Earl Brewer [L].

Virgil was a reserved, thoughtful, wonderful human being and another pioneer and legend. He trained at Johns Hopkins and settled in Los Angeles at USC and the Children's Hospital where he established what became a premier program. Virgil spoke softly and endeared himself to everyone who had the privilege of knowing him. He was always positive in his reaction to ideas. I never heard him speak ill of anyone, even when a person had really pulled a sneaky end-run around him or one of his many successful projects.

##### Dr. Sydney Stillman, Boston, Robert Breck Brigham Hospital, Harvard Medical School

Sydney was a distinguished adult rheumatologist at the Robert Breck Brigham Hospital in Boston, where he was medical director, and had pioneered a clinic for children with arthritis on a limited basis. He soon became president of the ARA. Sydney, who was older than the rest of us, became everyone's Uncle Syd. He was a gentleman in every way with a wonderful sense of humor that diffused many a tense situation. Syd was not only my professional hero but also a mentor as time progressed.

##### Dr. Jerry Jacobs, New York City, Columbia College of Physicians and Surgeons [figure 5]

Jerry Jacobs was another legend in pediatric rheumatology and an excellent pediatrician at Columbia in New York City. Jerry trained at Columbia and started a clinic at Babies Hospital. The entire room knew when Jerry entered. He was larger than life. Every event was a crisis to be solved. He had a heart of gold, but as a card-carrying-tough New Yorker, it was sometimes hard to find. Jerry was almost always the last to agree on any decision of our early collegial group and offered constructive opposition that became important to making our work relevant and credible. Ilona Szer and Yukiko Kimura in the pediatric rheumatology online journal gave a wonderful summary of Jerry and his work [3].

## Eleven pivotal events in the development of pediatric rheumatology

### I : Dr. William Clark and the March Of Dimes (MOD)

Dr. William Clark, Medical Director of the MOD, was a dynamic, maverick leader of rheumatology who was restless to take rheumatology to the next level of organization and success. Bill was a professor at Case Western Reserve when he accepted the challenge from Basil O'Connor and the MOD. Joe Levinson remembers that Bill visualized a three-tiered approach to childhood connective diseases. 1. Case finding with primary treatment facilities to be established in local communities at evaluation centers close to where patients live. 2. Special Treatment Centers would provide more expert and multidisciplinary services at academic institutions and would relate to the third tier. 3. Research Centers could be in the same center as the special treatment centers.

Bill was later the editor of the prestigious Arthritis and Rheumatism journal and led the merger of the ARA with the Arthritis Foundation. When he was president of the AF, Bill created the highly successful Arthritis Health Professions Section. Bill Clark knew that something had to be done about the care of children with arthritis. He was interested in our program of strengthening muscles and continued active exercise and activity because of data showing that enforced rest results in atrophy and weakness, not improvement. He agreed with us. This concept was discussed at the Vanderbilt meeting that he organized in Nashville in 1959.

### Vanderbilt meeting – 1959

The host for the meeting was Dr. Amos Christie, a revered Chairman of Pediatrics at Vanderbilt Medical School. Invited distinguished rheumatologists were Drs. William Clark, Howard Polley of the Mayo Clinic (a pioneer in the use of cortisone), Walter Bauer of Harvard and the Mass General Hospital, Joseph Bunim, Director of the National Institutes of Health, Charles Short from Harvard, Charles Christian, John Calabro, and a few others whose names escape me. Most were revered leaders of rheumatology. I was the only pediatrician present except for Dr. Christie. My assigned roommate was Chuck Christian who had just finished his fellowship at Columbia College of Physicians and Surgeons with Charles Ragen, another legend. Chuck had just joined the faculty at Columbia. He was an incredibly able young adult rheumatologist. We became friends over the years, and his help later was quiet but effective.

The discussion was heated. I was the only one who felt that these children must not be put to bed. The traditional thinking at the time was to put adults and children to bed in hospitals for as long as their health insurance lasted. Dr. Polley was so persuaded about the usefulness of bed rest that he said that he would return to the Mayo Clinic and put a few children with JRA to bed for six months and show the improvement in their joint X-rays. I heard later that he never was able to put any children to bed because pediatricians there were opposed to that practice.

Another belief of the adult group was that children with arthritis were a medical curiosity and were extremely rare. My thesis to them was that if meaningful care or facilities were available to the children, the parents would bring them. I suggested that cases of children with arthritis were not rare at all.

Dr. Christie, our host, then rose and spoke to the group for the first time. "I am not a rheumatologist, but as a pediatrician I know that you must not put these children to bed." He then left us. The effect was powerful. This to me was a pivotal meeting for pediatric rheumatology.

### Warm Springs meeting 1959 – Basil O'Connor MOD

**Figure 7:**
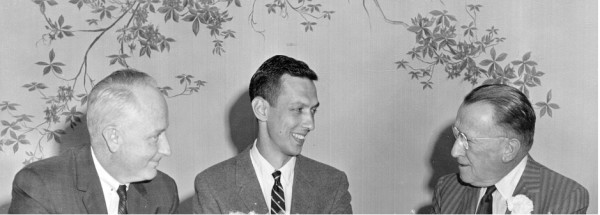
Dr. Ralph Block [MOD], Brewer, and Basil O'Connor, Founder March Of Dimes

The next pivotal meeting was at Warm Springs in 1959 with Basil O'Connor [figure 7], the respected founder of the MOD. The meeting was again small and was at President Roosevelt's Warm Springs retreat near Calloway Gardens in Georgia. I don't recall the total group beyond Bill Clark, Basil O'Connor, Ephraim Engelman, a rheumatologist from San Francisco, and myself. It was a powerful and historic meeting place. Basil O'Connor told the story of the early plans for polio care and research that took place in the very room where we sat. He felt that we should not be shy about moving forward.

Mr. O'Connor told us the story of the last days of President Roosevelt at Warm Springs before his death. It was a moving experience for all of us and gave me a sense of destiny for our new efforts. With Basil O'Connor's approval, Dr. Clark quickly moved to establish Special Treatment Centers for JRA under the auspices of the MOD.

### March Of Dimes, William Clark, Special Treatment Centers 1960

Texas Children's Hospital was among the first centers approved by the MOD. Joe Levinson in Cincinnati and John Calabro in New Jersey were directors of other funded centers. Other centers were added later. Our center's funding allowed hiring Mrs. Elizabeth Barkley as our physical therapist, and purchasing needed equipment. In 1962 further funding allowed me to become a half time faculty at Baylor and Texas Childrens Hospital. Soon thereafter there was a transition of funding support from the MOD to the Arthritis Foundation as the MOD felt more at ease with supporting neonatology programs under Virginia Apgar as their major funding interest. Bill Clark transferred our movement to the Arthritis Foundation successfully with the help of the MOD.

### My transition to Texas Childrens Hospital, Baylor College of Medicine, and Kelsey Seybold Clinic in 1961–1962

A major problem with the success of our fledgling and unsophisticated program was that I could only devote one full day each week to it. Our commitments now exceeded my time limits. Dr. Blattner and Dr. Mavis Kelsey then engineered a complex arrangement that brought me back to Houston full time. I remain grateful to both. Dr. Blattner obtained a grant from the MOD for a half time salary. This allowed me to be Assistant Professor of Pediatrics at Baylor and Director of the burgeoning program of the clinic. It also provided me with time in the lab for research projects. I had worked my way through college and medical school working in laboratories. Dr. James Kemper and Dr. Alfred Leiser also were crucial to the success of our program.

Dr. Mavis Kelsey, also a mentor and friend, is a pioneer at our Texas Medical Center. He founded his private clinic in 1949 after training at the Mayo Clinic. His dream was to build a large, multidisciplinary clinic. He did. The clinic is now the largest in Houston with over 300 doctors and 20 branches. In 1961 his clinic had six or seven doctors, and all were internal medicine specialists, mostly trained at the Mayo Clinic. He wanted to expand the scope of the clinic and invited me to found and chair the pediatric department. I accepted. He paid the other half of my salary. I arranged my time in the early days by working in the lab at Baylor in the Microbiology Department, and at the TCH clinic in the mornings and at the Kelsey Seybold Clinic (KSC) in the afternoons. My role was to build a pediatric practice at KSC (Kelsey-Leary Clinic in the early days). There are now 60 or more pediatricians in the clinic spread over 21 or more branch clinics. I was chair for over 20 years. Both programs became more successful than I ever imagined. Juggling time requirements did not end until I retired in 1990.

### II : ARA-JRA SUBCOMMITTEE 1963

Marion Ropes, then President of the American Rheumatism Association (ARA), appointed J. Sydney Stillman, Boston, to be chairman of the first Juvenile Rheumatoid Arthritis Criteria Subcommittee to study classification. I was named chairman in 1964 and served until 1977 when James Cassidy, Ann Arbor, assumed the chair.

**Figure 8:**
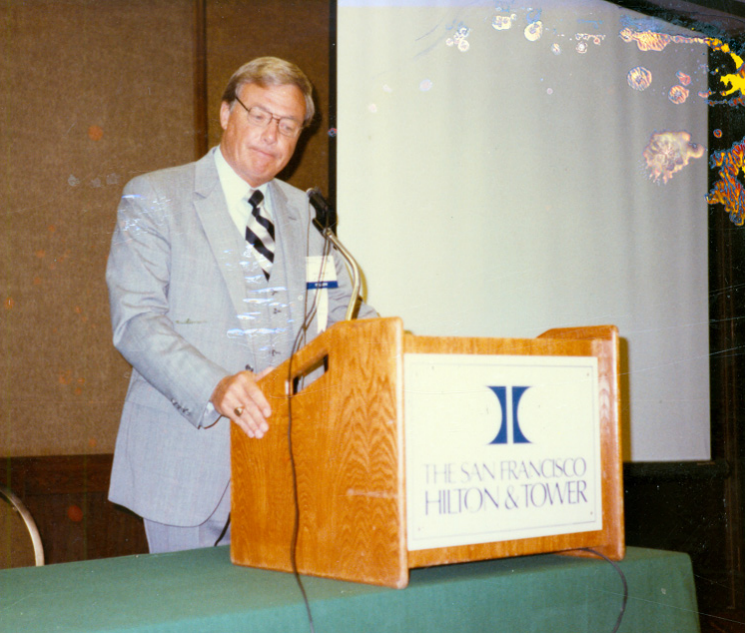
Dr. Jack Bass.

**Figure 9:**
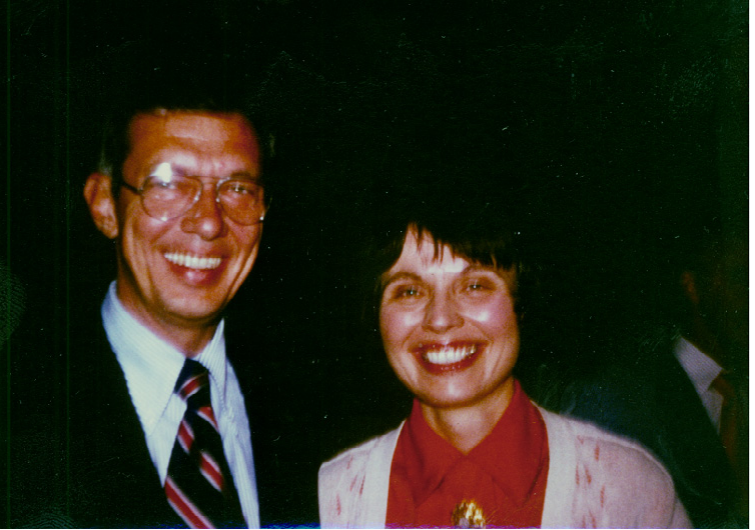
Dr. Jane Schaller [R] and Dr. Earl Brewer [L].

The other members of the original committee were Chester Fink (Dallas), William Gibson (Columbus) 1965–68 followed by Jack Bass (Columbus) 1968- [figure 8], Jerry Jacobs (New York City), Milton Markowitz (Baltimore and Hartford), William Reynolds (ex-officio, New York City), Jane Schaller (Seattle) [figure 9], J. Sydney Stillman (Boston), and Stanley L. Wallace (ex-officio, Brooklyn).

The appointment of a subcommittee for was a truly pivotal event for pediatric rheumatology. Marion Ropes was a Professor of Medicine at the Massachusetts General Hospital and Harvard Medical School and represented the most conservative element of the adult rheumatology world. Her friend, Sydney Stillman, Director of the Robert Breck Brigham Hospital rheumatology program, also at Harvard, persuaded her that studies of children with arthritis needed to be done and that a standard classification was needed to allow studies to be performed. Most adult rheumatologists at the time believed that children with JRA were a medical curiosity and rare in frequency.

Milton Markowitz was a mentor to the group and was older. He was at John Hopkins during this time. He soon went to the University of Connecticut as Chairman of Pediatrics and later as Dean of the medical school. Mark was a respected authority on rheumatic fever. Like Syd Stillman, he was a consensus builder, who had a wonderful sense of humor. Mark was raised in New York City and believed that some of the nicest people in the world were those who survived the streets of New York as youths and were still kind and considerate people. He was that person.

William Gibson was a member for a few years at the onset of the committee's work. Bill was a pediatrician at Ohio State Medical School in Columbus. He was articulate and aggressive in his approach to life. Some of us had strong opinions about life and the criteria. Bill and Jerry Jacobs had several explosive verbal encounters in our deliberations. Jack Bass replaced Bill on the committee in 1968. Jack also was a pediatrician in Columbus. He was a larger-than-life person, athletic in build, booming baritone voice, and was our bull-in-a-china closet member. As I remember, Jack played basketball at the University of Missouri and had an athlete's keen sense of competition. He was devoted to children with arthritis and always fulfilled his responsibilities in a timely fashion.

Jane Schaller was the youngest member of the group and became another pediatric rheumatology legend. She was from the University of Washington in Seattle and later became Chairman of Pediatrics at Tufts Medical School in Boston. Jane trained in the United Kingdom with Eric Bywaters and Barbara Ansell, well known and revered pioneers of rheumatology in the UK. Jim Cassidy, Virgil Hanson, and Chester Fink also spent time with Eric and Barbara. Jane was an assertive and able person. Her smile would light the room and her thoughts were penetrating and insightful. I was chair, coordinator, facilitator, persuader, and motivator of the group and was at Baylor and Texas Children's Hospital in Houston. William Reynolds and Stanley Wallace were ex-officio members from the ARA, and these members changed over the span of years of committee work.

The committee from the beginning formed a collegial spirit to develop a classification of JRA that would allow investigators to perform studies on uniform populations and provide structure for diagnosis. The committee took three approaches serially: (1) The committee exercising clinical judgment and experience, wrote a tentative criteria with exclusions to be tested. (2) A survey of clinical manifestations of 249 JRA patients was taken. (3) A prospective study of patients involving 135 JRA children and 100 non-JRA children with rheumatic complaints was done.[4]

#### (1)Classification I: empirical JRA definition with manifestations

The committee, all experienced in the care of children with JRA, developed 11 clinical manifestations with arthritis as a necessary component. Each manifestation was carefully defined. Exclusions were included. The original classification items to be tested were polyarthritis (two or more joints), monarticular arthritis, rheumatoid rash, rheumatoid factor, iridocyclitis, cervical spine involvement, pericarditis, tenosynovitis, intermittent fever, morning stiffness, and subcutaneous nodules. This grouping was termed Classification I. The committee felt that the clinical manifestations of JRA were so varied that exclusions were necessary for a valid classification. The exclusions were detailed and numerous.

Our collegial experience was that polyarthritis (two or more joints) present for three months or longer would allow a diagnosis unless an exclusion condition was present. Polyarthritis present for six weeks or less with one of the defined manifestations would allow diagnosis unless an exclusion diagnosis was present.

Spirited discussions were the hallmark of our first project together. We all felt the need and pressure to complete our task successfully. We knew that this would be our first, last, and only opportunity to move our cause forward. Our meetings were often in conjunction with other pediatric or ARA meetings. As I remember, we usually paid for our own expenses. We had a limited budget.

Even though detailed descriptions were made of the prominent manifestations, the inclusion of so many exclusions created the criticism that the classification was simply an exclusion diagnosis of chronic inflammatory arthritis to be classified when all other diagnoses were eliminated. We called this effort, Classification 1.

One has to remember that there was no unanimity on what JRA was. We carefully read Dr. George Frederic Still's classic description of what became Still's Syndrome from the Great Ormond Street Hospital in London. His first paper was for his dissertation at Cambridge in 1895, and his second paper was from the Medico-Chirurgical Transactions, London, 1897. [5] Jane Schaller found his original paper in the archives of the London hospital at a later date. **The thrust of our work was to develop a classification that would allow researchers everywhere to study children with JRA and obtain results that others would understand and accept**.

I remain fascinated by the many intellectual discussions regarding why we were NOT developing a diagnostic criteria, but rather a classification of JRA for researchers. The discussions were passionate. I pointed out that no matter what we wrote, physicians would use the classification as diagnostic criteria. In fact, that is exactly what happened.

The importance of diagnostic criteria versus classification discussions was relevant. We concluded that our further studies would try to develop criteria that would have high specificity for JRA, even if the sensitivity were low to help researchers who needed to eliminate other diseases by specificity data and not exclusions. My personal opinion was that physicians caring for JRA children needed nail-in-the-wall criteria that would aid in treatment decisions and diagnostic decisions. The retrospective survey was the initial move to ascertain if we could establish a time frame for earlier diagnosis. Six months was the time range for the adult RA criteria. We hoped to reduce that time frame with the initial review as well as verify that our clinical experience had validity.

#### (2) Retrospective survey of JRA patients

We concluded that we must not report to the ARA a compilation of eleven learned manifestations of JRA with no substantiating data and pass muster with our charge. Therefore, our next phase was to ascertain how soon the eleven items occurred in children with JRA diagnosed by experienced physicians. We needed to know how soon JRA could be classified after clinical onset. We also would learn whether data would validate our clinical judgment and experience. There was considerable discussion about creating a self-fulfilling classification. We addressed this legitimate concern with the second and third phases of our project.

The directors of nine clinics completed a retrospective survey to ascertain when the 11 clinical manifestations listed by the committee **first occurred **in their patients. All patients included had to have arthritis as a manifestation and were believed to have JRA by the physician. The participants were James Cassidy, Chester Fink, William Gibson, Robert Hill (Vancouver), Ralph Jacox (Rochester, NY), Joseph Levinson (Cincinnati), Milton Markowitz, and Jane Schaller. Benjamin Duran, Ph.D. (statistician, Houston) and I analyzed the data and participated in the data collection.

Of the 249 patients analyzed, 214 children would have been diagnosed in six weeks or less in terms of classification I; Nineteen additional patients would have been diagnosed within three months. Sixteen patients would not have been diagnosed by three months (6 percent error rate). Criteria I successfully identified 94% of JRA patients within three months. The sixteen patients not diagnosed had manifestations of disease other than arthritis in the first three months. By one year, ten of these patients developed arthritis, and six patients had arthritis after one year.

#### (3) Prospective study: computer analysis of JRA and Non-JRA patients with rheumatic complaints

The next necessary step was to design and perform a prospective, comparative study of JRA and non-JRA patients with arthritis. Our hope was that we would find that a classification could be developed from the database to allow flexibility and accuracy for differing needs of researchers. We also wanted to develop a general classification that would possess acceptable sensitivity and specificity to satisfy needs of researchers and practitioners.

Two hundred thirty-five patients (135 JRA and 100 non-JRA) from 16 centers were examined by center directors, or designated associates, during a specified nine-month period and examined again four to six months later. All patients with rheumatic complaints seen by the designated examiner were included. Patients had to be new to the examiner, but not necessarily a new JRA or non-JRA patient with rheumatic complaints. A detailed protocol was observed

Sixty-six different clinical manifestations and 23 laboratory items were recorded. Arthralgia was not included. The examiner made a diagnosis of JRA based on his/her own experience. There were 33 separate diagnoses in the 100 non-JRA patients. The largest number (17) had rheumatic fever.

Benjamin Duran then performed the statistical analysis. He was a joy to know, and it was a pleasure to work with him. The data was massive, and we programmed the data for exhaustive cluster analysis. Remember that we were working in the 1960's – the dark ages of computers. The computer with which we worked was housed in a huge, air-conditioned room and required enormous, tender, loving care.

The basic structure of the study remains a classic endeavor to me. On a prospective basis, we enrolled all patients with rheumatic complaints referred and examined by 18 center directors during a nine-month entry period. The patients had to be new to the examiner, but not new to rheumatic complaints. The design was close to the reality of pediatric rheumatology practice and made the results more relevant. I was delighted that the chance distribution was 135 to 100 JRA vs. non-JRA.

Analysis of the 89 items (66 clinical and 23 laboratory) presented a challenge. The chief problem was to reduce the large number of clinical manifestations in such a way that would be compatible with clinical judgment. A cluster analysis technique, which made use of the qualitative nature of the data (presence/absence data), was used to cluster similar (correlated) items. This method of analysis did not yield better results than those obtained by subjectively forming clusters of items.

Sensitivity and specificity of 89 individual items were used to arrive at the classification that we could recommend for approval. **Sensitivity **was defined as the percent of JRA patients having a particular manifestation present. **Specificity **was defined as the percent of non-JRA patients having a particular manifestation absent. For example, polyarthritis occurred in 108 of 135 JRA patients and 40 of 100 non- JRA patients. The sensitivity was 80 % and specificity was 60% for this item. Unfortunately no individual sensitivities or specificities yielded a 95% or better result.

We examined whether time from onset of disease could be helpful in early classification. We were surprised to find that 3, 6, 12 weeks and beyond yielded no increase in sensitivity or specificity. Even at 12 weeks or longer from disease onset 73% of JRA patients remained along with 41% of non-JRA patients. Counter to our collective, intuitive, clinical belief, 19% of the non-JRA patients remained after 3 months of disease. This meant that duration of disease beyond 12 weeks in itself was not helpful in the absence of exclusions.

We exhaustively examined combinations of items. Several hundred combinations were considered in our effort to find a significant relationship between JRA patients and controls. None reached 95% sensitivity or specificity. Symmetrical joint involvement did reach 70% sensitivity and 61% specificity. We attempted to show whether a certain number of joints would increase selectivity. To eliminate all but five percent of non-JRA patients would require 11 joints or more in the JRA population. The yield was too low to be effective as a classification.

This study did not provide any diagnostic help with monarticular arthritis. **Most children added more joints after a few months. **In a later study we classified pauciarticular arthritis as a separate subtype.

Many clinicians at the time felt that pericarditis was a definite item for classification. The data showed that there were more non-JRA children (five) than JRA children (four).

Fever also is an important component of JRA. Many of us had the mistaken idea that intermittent fever that lasts longer than six weeks is virtually diagnostic of JRA. Seven percent of JRA children had intermittent fever longer than six weeks, but four percent of non-JRAs also intermittent fever lasting longer than six weeks. Twenty-seven percent JRA and 28 % and non- JRA had lymphadenopathy. Of interest is that four JRA and no non-JRA children had epitrochlear nodes. Iridocyclitis occurred in four percent of JRAs and one non- JRA. This was an example of low sensitivity and high specificity.

Subcutaneous nodules occurred in six JRAs (five had a positive rheumatoid factor). Two non-JRAs with subcutaneous nodules had a negative rheumatoid factor.

Laboratory and x-ray data were not done in the same labs and could not really be analyzed statistically. Consistent tests were not done in each center.

Classifications II and III represented inclusion of all items of classification plus contracture of a joint and subjected the nine items to cumulative sensitivity and specificity calculations. The committee continued its work to validate and further refine it. We added subtypes: Systemic JRA, Pauciarticular JRA, and Polyarticular JRA. Cassidy, Schaller, and others established the relationship between iritis and Pauciarticular JRA.

The next project was to validate the newer data. [6] We used Joe Levinson's data at Cincinnati under Jim Cassidy's leadership. It was at this point that we used the new skills of computer technology with Jim Fries of Stanford Medical School. One memorable meeting took place at Joe Levinson's mountain home in Jackson Hole, Wyoming. Joe's house had large picture windows that provided a post card view of the Grand Teton mountain range. We shared bedrooms, and we made Jerry Jacobs and Chet Fink sleep in the same room because they snored so loudly. Jim Fries came to our meeting and showed off his new technology by Internet connecting to his home computer at Stanford in California. He showed us the capacity of analyzing data from several centers and joint meetings. It was a new world.

Joe and Jim were the leaders in the next phase of the criteria project, and the reader is directed to their recollections and data [7]. We were fortunate to have such able physicians as Jim Cassidy and Joe Levinson.

### III : publication of JRA book 1970 [8]

#### A. First edition

Mark Markowitz was a dedicated member of our JRA Subcommittee. He was probably the most prestigious member along with Sydney Stillman. Mark was well known for his studies with the streptococcus and rheumatic fever. He worked for several years in Israel in a lab with Ruth Kuttner. At Johns Hopkins he was a contributor to the Pediatric Clinics of North America published by W. B. Saunders & Company of Philadelphia. They were the largest publisher of medical books at that time in the 1960's. The series of books were classic. Mary Ellen Avery's classic Neonatology books came from this series and other authors' such as Mark and Ruth's book on rheumatic fever became classic books.

Mark and Alexander Schaeffer kindly asked me to write a book on JRA for the series. The other authors were well-known, well-published people. I was not. The opportunity was incredible. No one in pediatrics had written a book on JRA at that time, and Mark and his publisher Saunders thought that it would help to bring children with JRA to the consciousness of pediatricians. It was literally the blind leading the blind to write the book. My savior was a then-new-book editor at Saunders, Buck Rowan. He worked with me to structure a book properly. Years later several authors for Saunders told me that they were told to read my book to see how a book should be done. The reason was that my book was Buck's first assignment at Saunders. He became Editor-in-Chief years later. I started the arduous task in 1968, working at home in the evenings from 10 pm to after midnight. I enlisted the help of Elizabeth Barkley, our physical therapist, Ed Singleton, head of radiology at TCH (Texas Children Hospital), Malcolm Granberry, our orthopedist, Dan McNamara, head of cardiology at TCH, and Sidney Cleveland, our psychologist.

As a sign of the era, we devoted 8 pages to aspirin, 17 pages to steroids, 9 pages to gold therapy, 6 pages to indomethocin, a paragraph each to chloroquine and cyclophosphamide. The data reported was by-and-large our own data. There were remarkably few studies in children, and we analyzed our own patient base for most of the data. Our exercise program, a fundamental base to our treatment, received 23 pages.

The book sold several thousand copies chiefly due to the subscription list of Saunders. I was amazed. I learned a great lesson here. Many of my colleagues spent large amounts of time on national speeches and local talks in the hospital such as grand rounds. These efforts are essential for the education of our students, residents, and others. But as a tool to spread the word nationally and internationally about children with arthritis, the printed word lasts many times longer than any speech.

I was dumbfounded in 1976 to find my book in Leningrad, USSR. A group of rheumatologists including myself were in the USSR as part of the newly implemented USA-USSR scientific cooperation. I was the only pediatrician in the group of 12 or 14. We went to the offices of Professor Igor Vorontsov, head of pediatrics in Leningrad. He was in charge of the health of about one million children. He was also a pediatric rheumatologist. Rheumatology was one of the strong specialties in the USSR, and he had a substantial center with twenty or more physicians and accompanying staff. He was slightly plump, jolly but soft-in-speech man with a playful look in his eye. He quickly perceived that our adult rheumatology colleagues outnumbered us. Igor motioned me to come to his private office. Remember, that this was the middle of the cold war. He smiled and reached to his large, office library shelf and pulled a copy of my book. He told me that he read from it daily. I, of course, didn't believe that, but we had no idea that Russians had access to any of our books.

#### B. Second edition of JRA Book [9]

In 1973 Buck Rowan and I persuaded Jack Handy, the editor-in-chief of Saunders and Alexander Schaeffer, the consulting editor, that a second edition was useful. Pediatric rheumatology was moving ahead, and I wanted to keep up the momentum of our efforts. Unfortunately. my work load and lack of free time peaked in those years, and the book took a back seat for a few years. There were a number of letters from Buck wishing for more of my time to work on the book. Elizabeth Barkley and I did publish our Parents' Manuel with Saunders in 1975. Saunders also wanted to be a part of Park City I.

Buck Rowan was promoted in 1976, and George Vilk became my assigned editor. There is a memorable letter in my papers from me to George telling him that the book would be finished in 1976 -ho,ho,ho. The chapters were slow to be finished. I settled on writing three evenings each week with the help of our new associate, Ed Giannini. We lined all four walls of our dining room with reprints in metal separators. My wife Ria's mother was appalled. We had many dinner parties in the formal dining room surrounded by thousands of pages of data and reprints. It is hard to remember pre-computer data file days now. Ed lived with us three days each week with a pull down bed in our library. He had to use the one bathroom upstairs in the children's bedroom area and shared it with three of our children. I still hear from them about Ed. He spent so much time getting ready that they routinely pounded on the door. The regimen that Ed and I established was effective, and we completed the book by 1980. Mary Cowell had become the assigned editor in 1978. Ed and I decided to add Don Person, a colleague, as an author to get help in completing chapters, and we all struggled to finish the task.

As an example of how far drug therapy had progressed in childhood arthritis in 12 years, the first edition devoted 42 pages to drug therapy, while the second edition devoted 104 pages with many more medicines added and some eliminated. The second edition was published in 1982 and was successful.

**Other landmark, published books in pediatric rheumatology**: John J Miller III published Juvenile Rheumatoid Arthritis in 1979. Barbara Ansell published Disorders in Childhood (1980), Cassidy published the first edition of Textbook of Pediatric Rheumatology (1982). Jerry Jacobs published Pediatric Rheumatology for the Practitioner (1982). Gershwin and Robbins published Musculoskeletal Disease of Children (1983).

##### Parts II and III will be published soon in Pediatric Rheumatology

## Conflict of interests

The author(s) declare that they have no competing interests.
